# 

**Published:** 1887-11

**Authors:** 


Send in Your Subscription for 1888.

See Prospectus inside for Account of Premiums.

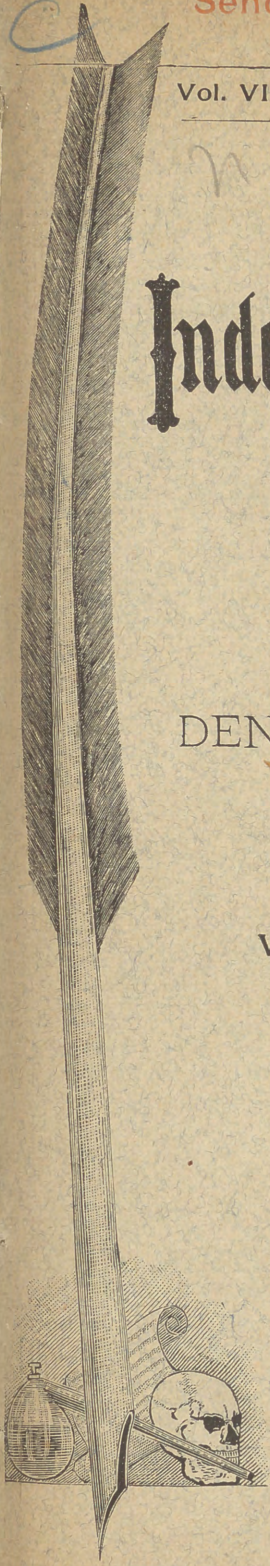

Vol. VIII. NOVEMBER, 1887. No. 11.

LIBRARY
INDEX
S.G.O.

THE

### Independent Practitioner:

### A MONTHLY JOURNAL

DEVOTED TO

### DENTAL AND ORAL SCIENCE.

---

**W. C. BARRETT, M. D., D. D. S.,**

EDITOR.

---

THE NEW YORK DENTAL JOURNAL ASSOCIATION,

PUBLISHERS,

No. 35 West 46th Street, New York.

AND

No. 208 Franklin St., Buffalo, N. Y.

**$2.50 Per Annum in Advance, - - - - Single Copies, 25 Cents.**

Entered at the Post-Office, Buffalo, N. Y., as Second-Class matter.

